# Conversion and the Real: The (Im)Possibility of Testimonial Representation

**DOI:** 10.1007/s11089-016-0692-6

**Published:** 2016-04-28

**Authors:** Srdjan Sremac

**Affiliations:** Department of Theology and Religious Studies, Vrije Universiteit Amsterdam, De Boelelaan 1105, 1081 HV Amsterdam, The Netherlands

**Keywords:** The real, Religious conversion, Canonical discourse, Testimonial representation, Lacan, Barthes

## Abstract

Although the spiritual vibration of conversion can be felt (by the curious outsider) through what conversion performers say in their testimonial discourse, what transforms the convert ‘on stage’ into a ‘new being’ and what is ‘the real’ (*le réel*) in conversion performance remain unclear. An important question in this connection is, What is ‘real’ in a conversion representation, both with respect to the convert’s interaction with the audience and to the construction of social reality? Following Lacan’s tripartite register of the imaginary, the symbolic, and the real, in this essay I argue that through testimonial discourse converts construct social reality as an answer to the impossibility of ‘the real’ in their performative discursive practice. In the first part, I question the constructed nature of testimonial representations—as well as some academic knowledge production that has governed conversion research in the last few decades—and how these representations encourage ‘outsiders’ to read the narrative repertoire as a negation or mirroring ‘the real’ of the conversion experience. In the second part, I apply Roland Barthes’ analytic reflections on photography to conversion research, especially the notions of the *studium* (the common ground of cultural meanings) and the *punctum* (a personal experience that inspires private meaning). This brings me to a number of theorists (mostly never used in the field of religious conversion)—Jacques Lacan, Roland Barthes, and Slavoj Žižek—who are important to the perspective that is developed in this essay.

Let’s get down, to the underground spiritual game.

—Fela Kuti, “Teacher don’t teach me nonsense”

## Introduction

Triggered and supported by my research on the role of conversion testimonial among recovering drug addicts, my work over the past several years in these critical endeavors has been specifically focused on the validity, reliability, objectivity, and other methodological challenges in the empirical study of religious conversion (Sremac 2013, 2014; Sremac and Ganzevoort 2013a, b). In this essay, I want to continue in this manner, focusing on Lacanian notions of the real in conversion phenomena and the (im)possibility of its testimonial representation. My use of the term ‘testimony’ comes from Ricoeur’s (1979) work on the hermeneutics of testimony, in which testimony is understood not only as cognitive assent but also as a narrative dialogue with the narrator’s audience and the divine (see Sremac 2014). The narrative capacity of conversion testimony—particularly since it belongs to the representative sphere—is directed towards an audience with the aim of persuasion. Stromberg (2014) argues that reframing a life story through a testimonial account is “a powerful means of persuading oneself and others that a genuine transformation has taken place” (p. 125). Testimony is a central ‘technique of the self’ (Marshall 2009) and the principal mode of creating a new identity and collective belonging. Conversion experience, regardless of its narrative configuration, must be ritually transformed and animated in order to contextualize the power of spiritual transformation. This ritual employment of conversion testimony requires transformation, consecration, or animation by the audience; it conveys experience, which can only be fully understood by those who have already gone through the same experience. Conversion testimonies, as they are retold orally and composed as autobiographies, become the paradigms by which converts interpret their lives and spiritual transformations (Rambo 1993, p. 158). In this sense, conversion testimony functions as a mechanism of reinforcement and commitment. It is a radical re-examination of one’s own life in the form of public-representation.

Following Lacan’s tripartite register of the imaginary, the symbolic, and the real, I argue that through testimonial discourse religious subjects construct social reality as an answer to the impossibility of the ‘real’ in their performative discursive practice. The testimonial order belongs to the *imaginary* register as a performative and imaginary frame, for example, the way the convert presents her or his spiritual transformation through testimonial performances and through identification (a mimetic movement) with others. The public aspect of this imaginary identification is perhaps a newly inscribed religious identity constructed through the gaze of others (Austin-Broos 2003, p. 2). Testimony is formed in the discourse of the other that becomes manifest in a public testimonial narrative (the communal-discursive construction of the ‘imagined self’). The *symbolic* order is the space of language and narratives and is passed through the filter of religious community; it is somewhat reminiscent of Stromberg’s (1993) ‘canonical language’. The symbolic order is considered to be the formative order of the convert; it is the ‘*sujet en proces*’ (Kristeva 1998) within which ‘canonical language’ can open itself to the sacred, the mystical, and the sublime. The symbolic order creates the narrative and culture in which the subject can express itself as a specific subject with its own identity.

The *real*, I argue, is to be taken not merely as something extra-imaginary but as a principal register that shapes the subject’s sense of reality and, at the same time, introduces the ‘constitutive lack’ that rotates conversion experience—the impossibility of the symbolic register to capture the fullness of a conversion lived experience in its deepest essentials and its totality. The real is, thus, ‘realized’ via the power of language that belongs to the symbolic and imaginary order as an attempt to represent the real. It is a supplement or the fulfillment of the lost or absent object. The testimonial repertoire is characterized by lack, dispersal, shattering, and a constant search for the missing or absent Other. It is the movement of spiritual desire that mobilizes this testimonial quest. This means that the real is the absent Other around which desire keeps rotating in movement, in action; the absence opens the space and potential for exposure to the real that can never be fully reached or reachable in itself. The real is the order, which cannot be articulated and represented in testimonial talk; at the same time, it infuses every aspect of the convert’s religious life. In a way, the real is inherent contradiction in the sense that it does not exist; it is “a hole in the symbolic order, but it is nevertheless described as a source of contingency” (Pirskanen 2008, p. 5).

The religious subject is (trans)formed in imaginary testimonial identifications with the other and experiences conversion through (canonical) language in relationship to the (symbolic) Other, but the real is the ‘constitutive lack’, the basis of both the *cause* and *object* of spiritual desire. These three orders of conversion cannot be entirely separated but rather are fundamentally interwoven. As I shall discuss, in testimony the real, imagination, symbolism, and desire emerge.

## Beyond the narrative-constructivist framework

Many social scientists today would agree that there is no such thing as ‘the conversion’, even though they believe it is possible to capture ‘the real’ of conversion and thus to show what conversion *really* is. Conversion was earlier understood in terms of an inner psychological transformation relative to a supernatural agency or to a change in sociocultural commitment and more recently as a political and ideological representation. Dealing with spiritual transformation is a tricky business; it is not an easy task. As Heirich (1977) points out, converts have developed arguments about the nature of the divine-human encounter, whereas social scientists have proposed a variety of social and psychological explanations of it (p. 653). Rambo (1993) expresses this doubt when he writes that “it is difficult to understand, predict, and control that which is generally invisible to the outsider, mysterious and sacred to the insider” (p. 24). In other words, it is not possible to access the core experience of conversion—the ‘real’ conversion. The words ‘predict’ and ‘control’ in Rambo’s quotation are particularly problematic since the conversion representation belongs to the subjective (often mystical) dimension of ‘truth telling’. These are destabilized categories that can barely be ‘predicted’, ‘controlled’ or ‘fixed’. In other words, the researcher is not in full control and cannot fully master or dominate the conversion experience. Converts usually argue that their experiences are opposed to academic knowledge production and only partially and obliquely accessible to scientific investigation. This can also be understood as converts’ “politics of authentication” (van de Port 2011, p. 16).

Yamane (2000), in his article “Narrative and Religious Experience”, addresses the current methodological difficulties with studies of religious experience, arguing that when we study the religious experience of the individual we cannot study ‘experiencing’, which is to say the religious experience in real *time* and *space*. Therefore, we must study retrospective linguistic representations of religious experience, including conversion. Yamane (2000) makes a clear distinction between *experiencing* and *an experience*: “While experiencing is a constant temporal flow from the standpoint of an individual and therefore *cannot be directly studied*, an experience is the intersubjective articulation of experience. . . . One cannot experience and reflect on experience at the same time” (p. 174). The articulation of an experience is temporally distanced from the experiencing itself because “all subjective meaning is constituted in retrospect through reflection, rather than in present moment of the lived experience” (p. 175). The interpretation and representation is therefore part of the experience in that any experience contains interpretative elements; it is an interpretative perception. What is presented is always some collection of the lived experience, or more precisely it is made up of the retrospective ‘interpretation-of-representation’. Ricoeur (1979, p. 144) argues that the testimonial account itself interprets and also gives to interpretation the contents of experience.^1^ Accordingly, conversion can only be studied on the level of its representation in testimonial form, and there is no direct purchase on the real experience. Although the real conversion is radically deducted and absent from any structure of representation, this does not mean that the via *negativa* of conversion is the best solution. It also does not mean we have to be silent about it, even though we are not able to directly grasp the ‘really real’ experience of conversion.^2^

The conversion narrative is the testimonial account of an interpreted experience of spiritual transformation; the experience comes down through a testimony (the imaginary frame), and the testimony is transmitted to the audience on the symbolic level or what Stromberg (1993) rather neatly calls a ‘canonical language’. The symbolic are the various representations and narrative codes that structure the testimonial apparatus. In this way, the conversion narrative is not the ‘real’ experience of spiritual transformation itself but a representation and articulation of the experience as it is stored in the memory of the convert. Therefore, an understanding of a conversion experience is possible only on the level of its narrative and symbolic interpretation. This requires critical narrative investigation of testimonial reliability (see Bruce 2006; Sremac 2013, 2014). However, with Staples and Mauss (1987, p. 138), I hold that conversion is fundamentally a subjective phenomenon (‘conversion-according-to-the-convert’), and thus only the subject is qualified to tell us who he or she really is.

Because of these methodological problems, conversion theorists have shifted their focus from ‘real’ conversion experiences to the narrative performance of account-giving. For example, current psychological and sociological literature devoted to the phenomenon of conversion has moved away from the *causes* and *consequences* of conversion and the stages of the conversion process, which have occupied most researchers’ attention for the last 40 years, to the more recent narrative-social-constructivist approach. The linguistic narrative perspective is becoming prevalent in contemporary research on conversion since Snow and Machalek’s (1983) first attempt to introduce a focus on language to the study of conversion. Narrative theorists argue that only through the testimonies of converts can conversion experiences be comprehended, and it is precisely for that reason that the conversion (narrative) corpus should be subjected to linguistic analysis.

Narrative conversion researchers consider conversion testimonies primarily as speech acts and analyze their structural/formal, rhetorical/symbolic features, and connection with the wider sociocultural context, including the specific religious tradition of the convert (Buckser and Glazier 2003; Giordian 2009; Gooren 2010; Harpham 1988; Hindmarsh 2014; Jindra 2014; Leone 2004, 2010; Marzouki and Roy 2013; Stromberg 1993, 2014; Zock 2006). The narrative-social-constructivist approach further helps us to observe how the subject begins to employ the specific rhetoric of the religious group, thereby incorporating into his or her life the language of transformation inherent to the particular group. The religious canonical language serves as a new frame of reference that has the potential to radically transform the worldview of the convert. The way in which people transform and articulate their spiritual evolution (reperform the conversion act) is often determined by *replication* and *repetition* of discourse. This regularized repetition is understood here in Deleuze's (1994) sense as a relationship and discursive behavior toward an event (conversion) that is not comparable or a counterpart to anything else. As such, repetition is experienced as an encounter with the impossible. Building on Kierkegaard’s ‘religious repetition’, Žižek (2001) holds that the act of Christian conversion is the proptotype of repetation as an attempt to repeat Christ’s own humilation. However, the effect of faith ‘grammar’ is most significant in the formation and shape of religious subjects. Conversion’s ‘autobiographical remix’ and its testimonial repetition not only reveals how religious identities are framed and created, it also shows how the converts make sense of themselves and how they construct meaning and interpret their lives and their world.

Narrative conversion investigations primarily understand conversion as a linguistic construction of self-performance whose focus is on interiority enacting, or the process of “giving an account of oneself” (Butler 2005). For example, Snow and Machalek (1984), Staples and Mauss (1987), Leone (2004), and Stromberg (1993) focus on the ‘lived experience’. Acknowledging a close connection between the conversion testimony and the biographical experience, they describe the socio-psychological functions that conversion testimonies fulfill in the biography of the converted person (Zock 2006, pp. 55–56) and underscore the socially constructed and communal character of conversion accounts. Staples and Mauss (1987), drawing on the work of Snow and Machalek (1984), argue that biographical reorganization is the marker and the only true indicator of conversion, which involves a change in one’s ‘universe of discourse’. Testimony means that a person’s communicative use of language (words, metaphors, symbolic interactions) undergoes a radical change as a result of the conversion experience in order to make sense of the self and the world (Staples and Mauss 1987, p. 135). Staples and Mauss (1987) take a functional approach to language and argue that a conversion narrative is not a reflection of some underlying change of consciousness but a tool to achieve self and communal transformation. They view conversion as a process that is “fundamentally one of self-transformation”. This “self-transformation is achieved primarily through language; [and] the convert plays an active role in his or her own self-transformation” (p. 146).

Stromberg (1993) follows Staples and Mauss’s approach to conversion. In *Language and Self-Transformation*, he provides a sophisticated and insightful analysis of the language and rhetorical techniques used by converts in their testimonial accounts. Stromberg starts with the assumption that conversion accounts are not a reliable source of information about the history of past events and experiences. The change efficacy of the conversion is not restricted to the original *event*.^3^ Stromberg argues that the process of telling and retelling conversion testimonies is an essential performance of faith, the framing of personal experience in canonical language, that the convert has fostered. Conversion, he proposes, is an ongoing process of identity formation and reality constitution that is intimately reflected in the language and discourse style of the conversion narrative. The ‘performance’ is a form of religious ritual activity in the present. Stromberg emphasizes, “It is through the use of language in the conversion narrative that the process of increased commitment and self-transformation take place” (p. xi). Stromberg’s study looks at the performance of conversion narratives and argues that the performance itself is central to the efficacy of the conversion. Because of this, he assumes that religious discourse represents ongoing efforts to resolve deep emotional conflicts and ambivalences in the converts’ lives.

Similarly, Hutchison (1963) argues that religious language is characterized as symbolic or expressive language used for “the purpose of total life orientation” (p. 13). The symbolic language serves as a link between a believer’s deep emotional concerns and the larger community. Stromberg’s (1993) main concerns are how the symbol system (social symbolic reality) used within a particular tradition can give the convert a sense of self-transformation and how self-understanding is socially constructed in the discursive communities of which the convert is a part. The discursive community also provides an outlet for the expression of the convert’s ‘transformative or redemptive self’, and it creates a discursively mediated environment in which a new model of narrative production emerges. As such, the communal discourse plays an important role in what I would like to call the ‘autobiographical remix of life’ and in narrative identity empowerment. The discursive environment makes it possible for converts to reinvent themselves through the production of new narrative identities. There arises a new rhetoric of conversion, implying and combining codes, symbols, and metaphors that specify well-established testimonial linguistic coordinates that manifest in the symbolic space.

Stromberg attempts to explain these transformative effects of conversion by building upon two root distinctions. First, he distinguishes between the *referential* and the *constitutive* functions of language as a component part of human communicative behavior. Stromberg argues that when converts share their testimonies they use a type of speech (‘meta-language’) that always comprises both the referential and the constitutive forms of communications.

Secondly, Stromberg distinguishes between two further subclasses of communicative behavior relative to conversion narratives: *canonical* and *metaphorical* language. Canonical language, which in Stromberg’s (1993) method is essentially referential, is “the most certain and unquestionable of meaning” (p. 12). Ganzevoort and Visser (2009) argue for a different understanding of the referential role of canonical stories. They describe canonical stories as culturally validated story structures, which means they regard the legitimacy an audience confers on a story a more important criterion than the referential role of the story. Canonical language refers to the religious context of meaning and becomes meaningful in a broader sense. It links canonical language directly with individual experiences (Popp-Baier 2002, p. 57). In this regard, canonical language fosters a change in the convert’s self-understanding and newly adopted religious role. This suggests that the conversion narrative is a practice through which converts seek to establish a connection between the language of their particular religious community and their own immediate situation. It enables the verbal expression of previously inaccessible desires while deepening the commitment to faith. The conversion testimony constitutes the narrator’s self-transformation. Metaphorical language (the symbolic frame) is about unfamiliar word usage undertaken to define and grasp the novel, the numinous (*das Heilige*), *mysterium tremendum* (Otto 1950), or the horror. In Lacaninan terms, it is the real as a radical and traumatic Otherness. In developing the conversion testimony, the *canonical* becomes constitutive (i.e., meaningful)—it becomes anchored in the details of the convert’s personal drama—and the *metaphorical* comes to be referential, ‘interpretable’. In this regard, conversion testimony is first and foremost a way of talking, a way of using language to make sense of spiritual transformation in an individual’s life.

To sum up thus far, conversion testimony as a ‘transformative practice of self’ is linked to the discursive communities that provide a subculture for understanding and explaining one’s ‘real’ experience and spiritual transformation. Testimony involves a willingness to have one’s life formed and transformed in and through the practices and patterns of canonical language. These patterns are immediate, localized, and authorized in the communal narratives and other religious symbols that enable converts to interpret their own experience. Communal discursive styles and regulations serve as guidelines that influence how a new experience is interpreted and significantly affect the interpretation of any other experience. The developed conversion testimonies must be embedded in, and constructed out of, a convert’s particular faith community and its formalized practices—that is, the specific grammar of faith and the pattern of its well-formed assemblage of belief and value system (Sremac 2014). Canonical language is transmitted and shared, it involves converts in particular symbolic coordinates, and it plays a central role in (trans)forming the converting subject. It reflects the convert’s ability to make use of established genre and discursive regimes in order to configure and constitute his or her life in culturally and religiously recognizable and acceptable patterns. In short, religious subjects gradually bring the lived experience of their lives into a resemblance of the core story of their faith community.

Following this theoretical framework, it is not entirely clear to whose reality or ‘reality’ the conversion testifies. Do testimonies constitute the reality of the transformative event itself? Who motivates the possibility of testimonial speech from the converted person? Is it possible to affirm the ‘real’ in the conversion phenomenon while allowing that such an affirmation can take place only through social and linguistic relationships? Should priority be given to the elusive and mysterious experience in the conversion phenomenon or to the social process? Can we reduce conversion ‘reality’ to speech embedded merely in relations and practices? Is conversion nothing more than a human construct? Is it simply the product of the personal relationships within a faith community, which has significant implications for the way in which converts form and organize their experiences and testimonies but possesses no intrinsic ‘ontological weight’ (Latour 2005)?

Conversion theorists have been concerned with the interplay between the linguistic practices of converts, but in their work there is a tendency to become preoccupied with the discursive details of variations in linguistic styles, semantic regimes, or faith grammar in a way that largely ignores other aspects of religious world-making embedded in lived experience. The conversion speech is never entirely comprehended by linguistic ‘grammar’. Drives, affects, fantasies, dreams, ecstasies, the sense and the nonsense (the absurdity) of conversion, hopes, desires, the unconscious, sensations, moods, and performative bodily actions are trans- and extra-linguistic experiences that constantly erupt into the symbolic/linguistic and the material^4^ order. Van de Port (2011) argues against the ‘discursive colonization’ of academic production, holding that constructivism, like other ‘isms’, is merely a different attempt to “uphold the truth of a vision against the constant intrusions of the Real; different designs to provide a particular world view with a sense of the real; different attempts to keep the-rest-of-what-is at bay to thus safeguard a particular reality definition—or invoke it to upgrade the persuasiveness, depth, credibility of that particular world view with a sense of a sacred ‘beyond’” (p. 29).

The reality of conversion, even though it is given in linguistic categories that belong to the symbolic register, cannot be reduced to a discursive statement of certainty. Converts use established discursive modes and regulations of conversion that help them to experience a spiritual transformation of reality—and that also seduce them into a particular relation with the signifying work of the ‘real’. In other words, converts can disorient themselves in the symbolic space through “modes of speech that have an impersonal nature” (Butler 2005, p. 52). The symbolic space is constructed through a mimetic activity, and the very possibility of discursive agency is delivered by the canonical language. Hindmarsh (2014), building on a Marxist conceptual framework (particularly Althusser’s notion of ‘interpellation’), shows how (conversion) language can be inscribed to people as interpellated by the dominant ideologies of cultural, political, or religious institutions. He argues that the narrative—or testimony in this context—becomes “not the expression of an individual point of view, but a site that registers the push and pull of social values as embodied in institutions and power structures” (p. 350). But the question is, What if the convert never really chooses this signifying grammar of faith? This signifying performance can indeed ‘alter symbolic reality’ by “transforming retroactively the signifying network which determines the symbolic significance of the ‘facts’. But here, signifying work ‘falls into the Real’, as if language could change extra-linguistic facts” (Žižek 2005, pp. 130–131). Beyond the shadow of reality, the conversion event points to the encounter with the sublime; the revelatory event as a dramatic reordering of the convert’s horizon of meaning. The real exists only in contradiction to reality, and it resembles the limits of language and presentation in general. This is precisely the most problematic aspect in academic knowledge production of the conversion experience and the convert’s public representation of it. I would like to challenge this rather limited constructivist understanding of conversion by adopting the Lacanian perspective of the real and problematizing the distinction between construction and the real experience in conversion scholarship.

## “The gods belong to the field of the real”^5^: Conversion and the quest for the real

According to Lacan, the real is the unknown that exists at the limit of the socio-symbolic universe (socially constructed representations), and it is a permanent strain with it. The real is the *cause* and *effect* of social reality, but it also undermines that reality. It occurs *prior* to symbolization and articulation, *prior* to the ‘materialization’ of the symbolic order (Žižek 2005, p. 192). In the Lacanian triad, the real is opposed to both the imaginary and the symbolic. The real is “the impossible” because it is impossible to imagine, impossible to integrate into the symbolic, and impossible to achieve (Lacan 1999). The real is something beyond comprehension in which language collapses. In short, the real cannot be summarized and totalized. For Žižek (1989), this negative sense of the real—though the real is not always a purely negative category—is “something that persists only as failed, missed, in a shadow, and dissolves itself as soon as we try to grasp it in its positive nature” (p. 169). Its “inertia blocks dialecticization, the ‘sublation’ in and through the symbol.” (Žižek 2005, p. 32).

In the context of this study, the real can be approached through testimonial repertoire; the real exists because it moves experience and it enters discourse as a sign, but it goes beyond symbolization and representation. That is to say, conversion is a matrix of representations, a matrix of symbols, narratives, imaginations, which are lived. The real is that which escapes symbolization and is outside of complete representation—that which cannot be articulated in testimonial talk. No matter how converts try to put their experience of spiritual transformation into language (to conceptualize it with the help of ‘canonical language’), there is always something left, something they have missed out on, something that goes beyond language and representation or what van de Port (2011) calls ‘the-rest-of-what-it-is’. In other words, there is always a deposit that cannot be transformed through language. The real goes beyond the subject’s discursive horizons; it is what forms and shapes our social reality, although it is expelled from it. Kay (2005) writes that no matter how subtle the real might be, it “insinuates its effects upon us; however negative, it remains a point of anchorage to which we are bound by enjoyment” (p. 5), and Žižek (2005) describes the real “*qua* drive . . . [as] the *agens*, the ‘driving force’, of desiring” (p. 192).

Thus, the narrative construction of conversion testimony is not the ‘reality’ but the analogical narrative that defines conversion. Conversion testimony is an interpretation of the real, a kind of second-hand experience that “fabricate[s] realities out of appearance” (de Certeau 1984, p. 186).^6^ I am not arguing here that conversion testimony is an empty performance, the false or illusionary representation of the social reality of the convert—as some theorists would argue—but rather that it is social reality itself that is framed in spiritual canonical language. Through testimonial performance and mediated canonical language, the religious subject is able to intimate and name the presence of divine power. The testimonial account creates an opportunity to build spiritual reality afresh. However, there is always something that is left out, non-articulated in conversion testimony, as a reminder of the real because the real is beyond interpretation as a missing point that cannot be fulfilled in the symbolic and imaginative realms. Precisely testimonial language is an attempt to interpret the real or to keep it real. Testimonial spectacle displays the real so as to provide only a reassuring image of it. In a way, it is “the surface of the real” (Barthes 2015, p. 32). The disturbing logic of the real is that it circulates in this ‘underground spiritual game’ as something that must be continually rediscovered and reinvented in order to avoid the “loss of reality” (Žižek 2005, p. 130). This is a rediscovering and remaking of the real in the reimagining, as a reflection of one’s self in the Other, not as mirrored reinvention but as personal encounter. It should be noted that for Lacan the real is unbearable and traumatic. The traumatic quality of the real ‘hits’ and ‘pierces’ our entire horizons of meaning, giving “a shock which dissolves the link between truth and meaning, a truth so traumatic that it resists being integrated into the universe of meaning” (Žižek and Gunjević 2012, p. 155).

Trauma precisely reveals how the real can never be completely absorbed and reflected into the symbolic, into social reality. A conversion experience can be understood sometimes as a traumatic experience, an event that transforms an individual’s horizon of meaning. As a traumatic event irrupts into our life and reconfigures the ways we see the world, the conversion event raptures the convert’s entire universe of meaning and reality and gives him or her an impossible encounter with the real. This brings me to Roland Barthes’ (1981) *Camera Lucida* as an important perspective on photography.

## The *studium* and the *punctum* of conversion

In his analytic reflections on photography, Barthes (1981) distinguishes two essential features of any photograph, the *studium* and the *punctum*. The field of cultural interest attaches meaning to the *studium*. The *studium* is the common ground of cultural meanings, the “contact arrived at between creators and consumers” (Barthes 1981, p. 26). The *punctum* (a Latin word derived from the Greek word for trauma), on the other hand, is a more personal experience that inspires private meaning, one that is suddenly recognized and consequently remembered. The *punctum* is something that ‘pierces’ and ‘wounds’ the observer (p. 146); it cuts the person “sharply, deeply, instantaneously” (Sontag 1977, 12). Barthes (p. 26) claims, “It is this element which rises from the scene, shoots out of it like an arrow, and pierces me.” The *punctum* is the symptom, relating to us on a deeply personal level.^7^ It is experienced personally and fervently, not just via formalized practices of cultural/conventional knowledge, which is the *studium.* It is hard to deny the existence of the *punctum*, whether it can be described logically or not. The *punctum* escapes symbolization and is beyond comprehension and representation; it avoids the grasp of verbalization. Or, to borrow Butler’s (2005, pp. 60, 135, 79) terms, the *punctum* is “inarticulable”, “unspeakable”, and “non-narrativizable”. It is therefore like Lacan’s real; the real that goes ‘beyond’ language and symbolization. In other words, Barthes’ detail that pierces and disrupts the *studium* (the symbolic order) of the photograph is precisely the encounter with the real. It concerns another system of non-linguistic representation, “not the *optionally* real thing to which an image or a sign refers but the *necessary* real thing which has been placed before the lens, without which there would be no photograph” (Barthes 1981, p. 76).

If we apply Barthes’ typology to the *studium* (the symbolic space) of conversion, it can be understood firstly as the canonical language of a faith community. A component of the *studium* and its direct application to conversion is the coding through which subjects are discursively ‘socialized’ in a faith community. The *studium* is, therefore, a kind of conventional knowledge that allows the spectator to discover the *Operator* (p. 26). The culture of the community provides discursive knowledge and regimes that allows the convert to discover the sacred or, in Barthes’ words, the Operator. Secondly, the *punctum* is a sudden *event* that pierces and transforms the convert’s relationship to reality, announcing the “new epochal disclosure of Being” (Žižek 2014, p. 31) with the “shakenness of being” (Zabala and Marder 2014, p. 2). The *punctum*, therefore, provides the ultimate frame of personal conversion, enabling the convert to experience the sublime. It is the emergence of a new ‘world’, the traumatic experience that disrupts the *studium* (the symbolic order) of the convert. The *punctum*, as an experience that goes beyond scripted common language and expectations, gives us some hints towards ‘the real’ as the promise of that, which resides outside of what is known, repeated, and enforced. In short, the *punctum* is the transformation of reality and of the past.

Beyond the symbolic appearance of the narrative work of testimony and its spiritual appeal, we should be aware that there is often a philosophical dimension; the subject forms part of a ritual or a principle of the real. Testimonies are an expression of a philosophical and theological maxim of life. The symbolic order functions as a framework of the real that enables entrusting oneself to the unexpected, uncharted way into the ‘traumatic’ reality in which the sacred comes. In this view, the ‘real’ experience of conversion creates a subject’s capacity to imagine and project beyond what can be experienced, narrated, or accomplished. In other words, the (im)possibility of representation of the real conversion opens up a space for the promised possibilities, which is the rest, the leftovers of the signifying action. It therefore becomes evident that this experiential level of the *punctum* requires a dramatic and ‘traumatic’ change in a convert’s personal life story. Van de Port (2011) has made this abundantly clear when he claims that the real goes beyond the “thoughts we cannot think, the feelings we cannot feel, the impulses upon which we cannot act if we are to remain who we take ourselves to be. It is ‘a kind of foreign body lodged inside’; an ‘alien wedge’ at the core of our being” (p. 253). And, “The total capture of experience in a structure of meaning—is an impossibility” (p. 256). The question that remains open is: Can we grasp the real, extraordinary, mysterious, and subtle dimension of being by constructing it backwards, as Eagleton (2009) suggests (p. 149)? If so, what would this discursive reconstruction look like?

## Footnotes

1 It should be noted that Ricoeur and Lacan probably cannot be harmonized on this point. In Ricoeur the representation always refers to something that can be represented. In Lacan the real is that which cannot be represented.

2 For quite some time there have been philosophical debates concerning the real and constructed nature of an experience and the articulation of the experience. I refer here to the long-standing debate between Katz (1977, 1983, 1992) and Hood (2001) on the role of language in mystical experience.

3 In Lacanian terms, the moving (transformatory) desire is not situated in the real (instead, it is in the symbolic and imaginary registers) but only keeps moving because of its direction towards its ultimate ‘real’ object.

4 The material/physical dimension of conversion is often a neglected area of study. It is important to investigate religious/spiritual everyday practices that involve, for example, doing, seeing, touching, tasting, and smelling. See Meyer’s (2014) material approach to religion.

5 Lacan (1981, p. 45).

6 Here we can benefit from Certeau precisely because of his applications of Lacanian thought to Christianity; the empty grave (the real) is the cause of the production of Christian *logos* in discourse and institutions.

7 Susan Sontag (1977) has described how seeing photographs of Bergen-Belsen and Dachau changed her life. Using almost ‘conversion paradigm discourse’, she said that photographs “divide my life into parts before I saw those photographs (I was twelve) and after” (p. 12).
